# Association Between the Ratio of Ovarian Stimulation Duration to Original Follicular Phase Length and *In Vitro* Fertilization Outcomes: A Novel Index to Optimise Clinical Trigger Time

**DOI:** 10.3389/fendo.2022.862500

**Published:** 2022-07-25

**Authors:** Xinyang Zhao, Xu Zhang, Shanshan Wu, Jichun Tan

**Affiliations:** ^1^ Center of Reproductive Medicine, Department of Obstetrics and Gynecology, Shengjing Hospital of China Medical University, Shenyang, China; ^2^ Key Laboratory of Reproductive Dysfunction Disease and Fertility Remodeling of Liaoning Province, Shenyang, China

**Keywords:** infertility, fertilization, gonadotropins, ovulation stimulation, menstrual cycle

## Abstract

The duration of ovarian stimulation which is largely dependent on the ovarian response to hormonal stimulation may influence *in vitro* fertilization (IVF) outcomes. Menstrual cycle length is potentially a good indicator of ovarian reserve and can predict ovarian response. Ovarian stimulation and the follicular phase of the menstrual cycle are both processes of follicular development. There is no published research to predict the duration of ovarian stimulation based on the length of the menstrual cycle. Our retrospective cohort study included 6110 women with regular menstrual cycles who underwent their first IVF treatment between January 2015 and October 2020. Cycles were classified according to quartiles of the ratio of ovarian stimulation duration to original follicular phase length (OS/FP). Multivariate generalized linear models were applied to assess the association between OS/FP and IVF outcomes. The odds ratio (OR) or relative risk (RR) was estimated for each quartile with the lowest quartile as the comparison group. OS/FP of 0.67 to 0.77 had more retrieved and mature oocytes (adjusted RR 1.11, 95% confidence interval [CI] 1.07–1.15, p for trend = 0.001; adjusted RR 1.14, 95% CI 1.09–1.19, p for trend = 0.001). OS/FP of 0.67 to 0.77 showed the highest rate of fertilization (adjusted OR 1.11, 95% CI 1.05–1.17, p for trend = 0.001). OS/FP > 0.77 had the lowest rate of high-quality blastocyst formation (adjusted OR 0.81, 95% CI 0.71–0.93, p for trend = 0.01). No apparent association was noted between OS/FP and clinical pregnancy, live birth, or early miscarriage rate. In conclusion, OS/FP has a significant effect on the number of oocytes, fertilization rate, and high-quality blastocyst formation rate. MCL could be used to predict the duration of ovarian stimulation with an OS/FP of 0.67 to 0.77, which provides a new indicator for the individualized clinical optimization of the trigger time.

## Introduction

In recent years, the prevalence of infertility has gradually increased, and assisted reproductive technology (ART) has emerged as the main method to solve intractable infertility. The menstrual cycle is a crucial clinical reference for female reproductive health; it repeats approximately 500 times over the reproductive lifespan, which lasts about 35 to 40 years (menarche to menopause) (1). The median menstrual cycle length (MCL) is 28 days, and most cycles are between 21 and 35 days in length. The menstrual cycle can be separated into two stages: the follicular or proliferative phase and the luteal or secretory phase. The follicular phase accounts for 84% of the variation in MCL (2), whereas the luteal phase is relatively constant at approximately 14 days (3). Epidemiological data demonstrate that menstrual cycles vary in length and regularity and are affected by age, ethnicity, body mass index (BMI), and behavioral, occupational, and environmental factors (4, 5).

Controlled ovarian stimulation (COS) is a key component of successful ART treatment which aims to achieve the synchronized development of multiple follicles in a menstrual cycle (6). Compared with traditional COS methods, personalized ovarian stimulation has a more positive effect on *in vitro* fertilization (IVF) outcomes (7, 8). Serum anti-Mullerian hormone (AMH) and antral follicle count (AFC) are the most reliable contemporary indicators currently employed to assess ovarian reserve before ovarian stimulation in clinical practice and are highly sensitive and specific in detecting the quantitative aspects of ovarian reserve, including ovarian responsiveness (9, 10). Moreover, MCL is potentially a good indicator of ovarian reserve; a short MCL of 21–27 days, compared with one of 28–31 days, is associated with lower AMH and AFC, reduced fecundability in natural cycles, and poor IVF outcomes (11). MCL correlates with both the quantitative and qualitative aspects of ovarian reserve and may be employed as an earlier, more subtle sign of ovarian aging relative to physiological age (12). In addition, studies have shown that MCL can predict low and high ovarian responses in ART (13–15).

Clinicians may struggle with the actual trigger time, such as the presence of large lead follicles with numerous small follicles at the same time. Several studies have investigated the effects of ovarian stimulation duration and gonadotropin dose on IVF outcomes, but the results are inconsistent (16–25). The process of ovarian stimulation is similar to the follicular phase of the menstrual cycle, and the duration of ovarian stimulation is largely dependent on the ovarian response to hormonal stimulation. We hypothesized that the MCL may be used to predict the duration of ovarian stimulation and that a more favorable IVF outcome would be achieved when the MCL and the duration of ovarian stimulation are within a certain quantitative relationship.

Our study aimed to investigate the effects of the relationship between the duration of ovarian stimulation and the duration of the previous menstrual cycle on IVF/ICSI outcomes and provide a novel index for controlling the duration of ovarian stimulation and optimising the clinical determination of trigger time.

## Materials and Methods

### Inclusion and Exclusion Criteria

**Figure 1:**
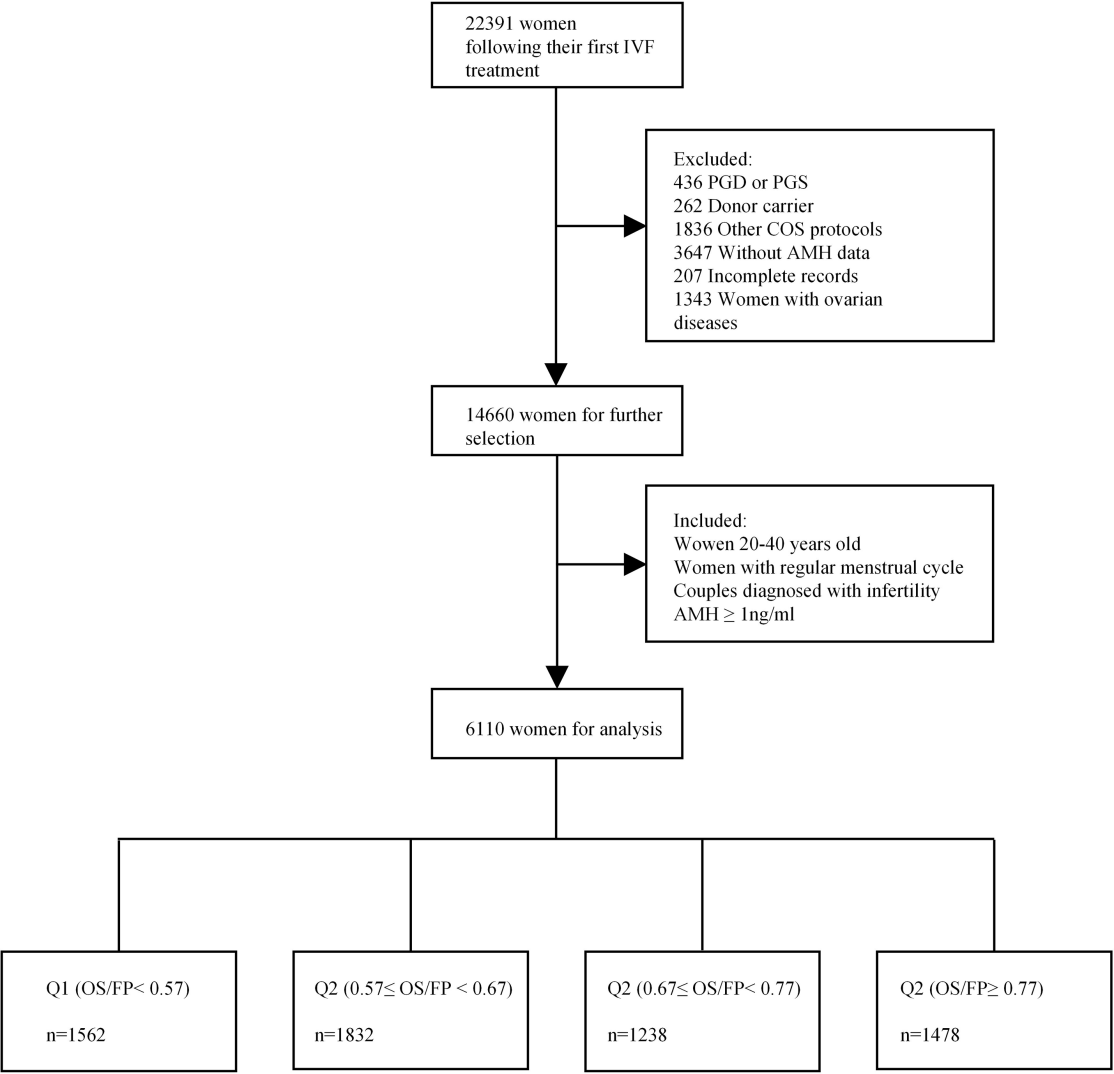
A flowchart of data preparation for analysis.

A total of 6110 women with regular menstrual cycles who underwent their first fresh IVF/ICSI cycle at the reproductive centre of Shengjing Hospital in Shenyang, China, between January 2015 and October 2020 were recruited in this study. The infertility diagnosis was assigned according to previously described definitions of the Society for Assisted Reproductive Technology (26). As shown in **Figure 1**, the inclusion criteria were as follows (1): women between the ages of 20 and 40 years (2); MCL between 21 and 35 days (3); couples diagnosed with infertility (failure to conceive with unprotected intercourse for 12 months or longer) (4); IVF/ICSI indications including unexplained infertility, tubal factors, or a male factor (including oligospermia, asthenospermia, or obstructive azoospermia); and (5) minimum AMH threshold of 1 ng/ml (We excluded women with low ovarian reserve with a cut-off of AMH <1 ng/mL for diminished ovarian reserve (DOR) which was consistent with studies reporting age-specific normal values (27, 28). We did not use DOR diagnosis by the Society for Assisted Reproductive Technology (SART) in our study as it has been shown that DOR is overdiagnosed in the SART reporting system (29).). Those with cycles involving donor oocytes, preimplantation genetic diagnosis, preimplantation genetic screening, or incomplete records were excluded. Women who were diagnosed with polycystic ovary syndrome, ovarian endometriosis, or other ovarian diseases were also excluded. Information on demographics, medical history, and clinical data were extracted from electronic medical systems. Our study was approved by the Ethics Committee of the Shengjing Hospital of China Medical University (2021PS001F), and the requirement of obtaining informed consent was waived owing to the retrospective nature of the study.

### Ovarian Stimulation and the IVF/ICSI Procedure

Prior to the IVF cycle, women who underwent ovarian reserve testing were allocated to one of three ovarian stimulation types as clinically indicated, which included the long-term, short-term, or antagonist protocol. The women’s serum oestradiol (E2), luteinizing hormone, follicle sizes, and follicle counts were monitored during ovarian stimulation. Subcutaneous recombinant follicle stimulating hormone (Gonal-f NGPEN, Merck Serono, Germany) or human menopausal gonadotropin (MENOPUR, Ferring, China) was administered with a combined daily dose of 150-300 IU. Ovulation was triggered with hCG (Crinone, Merck Serono, Germany) when three or more follicles reached ≥ 18 mm in diameter. Thirty-six to thirty-eight hours after the hCG trigger, a transvaginal ultrasound-guided oocyte aspiration was performed. The oocytes were then retrieved and fertilized using IVF/ICSI procedures based on the semen quality. One blastocyst or two cleavage embryos were transferred on the third or fifth day after fertilization. Progesterone was administered for luteal support after oocyte retrieval.

### Outcome and Embryo Quality Assessment

The numbers of retrieved mature oocytes (metaphase II, MII), oocytes with two pronuclei, and good-quality embryos were evaluated by embryologists. The fertilization rate, defined as the number of oocytes with two pronuclei divided by the number of oocytes inseminated, was determined 17–20 hours after insemination. On day 3, the Peter scoring system (30, 31) was used to assess the quality of the embryos based on the size, shape, and fragmentation of the blastomeres. Embryos with 6–10 cells, even size, regular shape, and < 20% fragmentation were considered good-quality embryos. At the blastocyst stage, the embryos were evaluated using the Gardner system (1): blastocysts were rated as grades 1–6 according to the extent of blastocyst expansion and hatching, and (2) further A–C scores were assigned to grade 3–6 blastocysts based on the number and cohesiveness of the inner cell mass and trophectoderm. A high-quality blastocyst was defined as having a grade ≥ 3BB on day 5 or ≥ 4BB on day 6. The proportion of blastocysts and high-quality blastocysts formed was evaluated.

We defined biochemical pregnancy as a serum β-hCG level > 30 mIU/mL on day 14 after embryo transfer. Clinical pregnancy was defined as the presence of an ultrasound-confirmed intrauterine pregnancy 35 days after embryo transfer. A miscarriage was the spontaneous loss of pregnancy before 12 weeks of gestation. A live birth was referred to a newborn delivered on or after 24 weeks of gestation. The primary outcomes were clinical pregnancy and live birth. Retrieved oocytes, fertilization rate, and embryo quality were considered intermediate outcomes.

### Data Classification and Statistical Analyses

The MCL was determined by calculating the average length of the menstrual cycle for the three months prior to the consultation without any hormonal stimulation. Follicular phase length was calculated by subtracting 14 days from the MCL. Patients were classified by quartiles of the ratio of the duration of ovarian stimulation to the follicular phase length (OS/FP) (**Supplementary Table 1**).

The demographic and clinical characteristics of the participants were reported using mean ± SD or percentages. Associations between categorical variables were analyzed using chi-squared tests or Fisher’s exact tests when one or more cell counts were ≤ 5. Comparisons between various variables and quartiles of the OS/FP were performed using the Kruskal–Wallis test, and the Bonferroni correction was used to account for the increase in type I error. Multivariate generalized linear models were applied to assess the association between OS/FP and IVF outcomes. A Poisson distribution with log link function was used to test the association between the numbers of total and mature oocytes, and a binomial distribution with logit link function was used for fertilization rate, quality of each embryo, and pregnancy outcomes. Overall linear trends were tested across quartiles using the classification number as a continuous variable. The odds ratio (OR) of fertilization, embryo quality, and pregnancy outcomes were estimated for each quartile with the lowest quartile as the comparison group. The relative risk (RR) for the numbers of retrieved oocytes and mature oocytes were estimated for each quartile with the lowest quartile as the comparison group. An RR or > 1 denotes an increase in the prevalence risk and odds of the target events as the ratio between the duration of ovarian stimulation and the follicular phase length increases by one quartile. Moreover, nomograms of fertilization and high-quality blastocyst were developed based on corresponding independent significant factors. Internal validation was performed using the bootstrap method. C-index and calibration curves were used to assess predictive and discriminatory capacity.

Covariates including age (years), BMI (kg/m2), smoking status, fertility status (primary infertility or secondary infertility), etiology of infertility (male factor, female factor, mixed factor, or unexplained infertility), AMH (ng/mL), ovarian stimulation type (long GnRH-agonist, short GnRH-agonist, or GnRH-antagonist), initial gonadotropin dose (IU) and insemination methods (IVF or ICSI) (32) were collected. The variables included in the final model were statistically significant variables (p < 0.15) of the outcomes or those with critical biological significance, such as age and BMI (33). We further assessed the effects of OS/FP on IVF outcomes across female age (≤ 30, 30–35, > 35) and ovarian stimulation types (long GnRH-agonist or GnRH-antagonist) using subgroup analysis. Statistical Package for Social Sciences software (SPSS, version 22.0; Chicago, IL, USA) was used for all statistical calculations. Nomograms were formulated using the package “rms” in R software, version 3.6.3. (http://www.r-project.org/). All reported p values were based on two-sided tests and compared with a significance level of 5%.

## Results

In this study, 6110 women who underwent IVF/ICSI treatment for the first time were included. Their mean age and BMI were 31.5 ± 3.9 years and 22.7 ± 3.3 kg/m2, respectively, and 180 (2.9%) women were obese (BMI ≥ 30 kg/m2) (**Supplementary Table 2**). A total of 2747 (45.0%) women had a history of pregnancy; 436 (7.1%) had a history of live birth; and 2019 (33.0%) had a history of miscarriage. The number of women with a university degree or higher was 2391 (39.1%). The majority of women had never smoked, and only 102 (1.6%) smoked before pregnancy. Secondary infertility was noted in 2424 (39.7%) women. Moreover, the following diagnoses were identified: female factor infertility, 3119 (51.0%); male factor infertility, 1276 (20.9%); mixed male and female factor infertility, 1506 (24.6%); and unexplained infertility, 209 (3.4%). IVF and ICSI were used for fertilization by 3831 (62.7%) and 2279 (37.3%) couples, respectively. A total of 2812 (46.0%) couples had embryos cultured to blastocyst stage. In this cycle, 2648 (43.3%) women had fresh embryos transferred, of which 1258 (47.5%) had clinical pregnancies and 942 (35.6%) had live births.

The clinical and reproductive characteristics of the women are shown in **Table 1** according to the quartiles of OS/FP. OS/FP < 0.57 had higher AMH levels, a longer MCL and menstrual bleeding duration than OS/FP ≥ 0.77. No differences were noted among the four groups in terms of age, BMI, fertility history, education, smoking, age at menarche, etiology for infertility, and fertilization method. In terms of ovarian stimulation, more women with OS/FP < 0.57 and OS/FP of 0.57 to 0.67 used the GnRH-antagonist protocol for ovulation. The largest proportion of women with OS/FP of 0.67 to 0.77 and OS/FP ≥ 0.77 used the long GnRH-agonist protocol for ovulation. OS/FP ≥ 0.77 had a higher initial gonadotropin dose, a higher total gonadotropin dose, and a higher duration of ovarian stimulation than OS/FP < 0.57. No significant differences were noted in good-quality embryos, blastocyst formation, clinical pregnancy, live birth rate, miscarriage, or ectopic pregnancy rate among the four groups. OS/FP of 0.67 to 0.77 had the highest number of retrieved oocytes (12.6 ± 7.1) and cleaved embryos (8.3 ± 5.0), OS/FP > 0.77 had the highest fertilization rate (70.4 ± 22.4) and OS/FP of 0.57 to 0.67 had the highest rate of high-quality blastocyst formation (36.6 ± 27.2) (**Table 2**).

**Table 1:** Demographic and clinical characteristics of all the participants (N = 6110).

	OS/FP quartile				*P*-value^a^
	Q1 (<0.57)	Q2 (0.57, 0.67)	Q3 (0.67, 0.77)	Q4 (≥0.77)	
N	1562	1832	1238	1478	
Age (years)	31.3 ± 3.9	31.5 ± 3.9	31.5 ± 3.9	31.7 ± 4.0	0.113
≤30, n (%)	664 (42.5)	739 (40.3)	518 (41.8)	599 (40.5)	
30-35, n (%)	666 (42.6)	799 (43.6)	517 (41.8)	585 (39.6)	
>35, n (%)	232 (14.9)	294 (16.0)	203 (16.4)	294 (19.9)	
BMI (kg/m2)	22.6 ± 3.2	22.7 ± 3.3	22.8 ± 3.5	22.7 ± 3.4	0.790
BMI (kg/m2) category^b^					
Underweight (<18.5)	113 (7.2)	135 (7.4)	95 (7.7)	118 (8)	0.423
Normal (18.5–24.9)	1092 (69.9)	1247 (68.1)	826 (66.7)	973 (65.8)	
Overweight (25.0–29.9)	291 (18.6)	357 (19.5)	256 (20.7)	318 (21.5)	
Obese (≥30.0)	41 (2.6)	48 (2.6)	43 (3.5)	48 (3.2)	
AMH (ng/ml)	4.1 ± 2.8	3.8 ± 2.6	3.9 ± 2.7	3.4 ± 2.2	<0.001
Gravidity					
0	872	987	692	812	0.622
1+	690	845	546	666	
Parity					
Nulliparous	1464	1702	1143	1365	0.411
1+	98	130	95	113	
Abortions					
0	1079	1201	832	979	0.159
1+	483	631	406	499	
Menstrual cycle length (days)	30.5 ± 1.8	29 ± 2	28.9 ± 1.5	27.4 ± 1.9	<0.001
Usual menstrual bleeding (days)	5.3 ± 1.4	5.2 ± 1.4	5.2 ± 1.4	5.1 ± 1.5	0.010
Age at first menstruation (years)	13.6 ± 1.2	13.6 ± 1.2	13.6 ± 1.2	13.6 ± 1.2	0.459
Duration of infertility (years)	3.7 ± 2.7	3.7 ± 2.7	3.8 ± 2.8	3.9 ± 2.8	0.104
Bachelor or above	613 (39.2)	751 (41.0)	486 (39.3)	541 (36.6)	0.084
Current Smoker	25 (1.6)	28 (1.5)	20 (1.6)	22 (1.5)	0.992
Fertility status					
Primary infertility	957 (61.3)	1077 (58.8)	752 (60.7)	900 (60.9)	0.444
Secondary infertility	605 (38.7)	755 (41.2)	486 (39.3)	578 (39.1)	
Etiology of infertility					
Female factor	765 (49)	947 (51.7)	635 (51.3)	772 (52.2)	0.421
Male factor	357 (22.9)	388 (21.2)	253 (20.4)	278 (18.8)	
Mixed factors	387 (24.8)	438 (23.9)	307 (24.8)	374 (25.3)	
Idiopathic	53 (3.4)	59 (3.2)	43 (3.5)	54 (3.7)	
Type of ovarian stimulation					
Long GnRH-agonist	601 (38.5)	898 (49)	723 (58.4)	974 (65.9)	<0.001
Short GnRH-agonist	34 (2.2)	91 (5)	54 (4.4)	64 (4.3)	
GnRH-antagonist	927 (59.3)	843 (46)	461 (37.2)	440 (29.8)	
Initial gonadotropin dose, IU	225.2 ± 53.6	228.2 ± 54.2	230.6 ± 55.3	233.2 ± 56.8	<0.001
Total gonadotropin dose, IU	1885.1 ± 602.1	2359.2 ± 827.5	2543.1 ± 764.5	2950.6 ± 879.7	<0.001
Endometrial thickness-HCG, mm	10.4 ± 2.7	10.2 ± 2.8	10.1 ± 2.7	10.0 ± 2.9	0.001
Duration of stimulation (days)	8.3 ± 1.3	9.9 ± 2	10.6 ± 1.3	11.8 ± 2	<0.001
Insemination technique					
IVF	963 (61.7)	1162 (63.4)	775 (62.6)	931 (63)	0.751
ICSI	599 (38.3)	670 (36.6)	463 (37.4)	547 (37)	

Values are n (%) for categorical variables and mean ± SD for continuous variables. SD, standard deviation; OS/FP, the ratio of duration of ovarian stimulation to follicular phase length; BMI, body mass index; AMH, anti-mullerian hormone; IVF, in vitro fertilization; ICSI, intracytoplasmic sperm injection.

Data on covariates were missing for BMI (n = 109), endometrial thickness-HCG (n = 99).

a P values were calculated from nonparameter test (Kruskal-Wallis) or χ2 test.

b BMI category (WHO classifification).

**Table 2:** Cycle Characteristics of all the participants (N = 6110).

	OS/FP quartile				*P*-value^a^
	Q1 (<0.57)	Q2 (0.57, 0.67)	Q3 (0.67, 0.77)	Q4 (≥0.77)	
Retrieved oocytes	11.4 ± 6.5	11.9 ± 6.3	12.6 ± 7.1	11.4 ± 6.3	<0.001
Fertilization rate, %	68.0 ± 22.5	68.7 ± 22.6	69.9 ± 22.4	70.4 ± 22.4	<0.01
Cleaved embryos	7.3 ± 4.6	7.7 ± 4.8	8.3 ± 5.0	7.5 ± 4.6	<0.001
Good-quality embryos rate, %	64.6 ± 31.8	65.8 ± 32.6	65.3 ± 30.8	63.7 ± 32.0	0.208
Embryos cultured past day 3^b^	6.7 ± 4.1	7.0 ± 4.2	7.3 ± 4.1	6.6 ± 3.9	<0.01
Blastocyst formation rate, %	46.8 ± 28.4	48.0 ± 28.6	44.7 ± 27.6	46.0 ± 30.2	0.067
High-quality blastocyst rate, %	35.8 ± 27.8	36.6 ± 27.2	33.3 ± 26.3	32.7 ± 28.0	<0.01
Fresh cycles with embryo transfer^c^	636 (40.7)	823 (44.9)	525 (42.4)	664 (44.9)	0.042
Number of embryos transferred	1.8 ± 0.4	1.8 ± 0.4	1.8 ± 0.4	1.9 ± 0.4	0.034
Clinical pregnancy	297 (46.7)	388 (47.1)	260 (49.5)	313 (47.1)	0.735
Live birth	233 (36.6)	272 (33.0)	204 (38.9)	233 (35.1)	0162
Spontaneous abortion before GW 12	32 (5.0)	59 (7.2)	33 (6.3)	43 (6.5)	0.420
Ectopic pregnancy	4 (0.6)	9 (1.1)	5 (1.0)	8 (1.2)	0.738

Data was described as mean ± SD or n (%). GW, gestational week.

^a^P values were calculated from nonparameter test (Kruskal-Wallis) or χ2 test.

^b^A total of 2812 participants had embryos continuously cultured to the blastocyst stage.

^c^A total of 2648 participants transferred embryos in this fresh cycle.

The effects of OS/FP on the number of retrieved and mature oocytes in the corrected multivariate model are described in **Table 3**. OS/FP ≥ 0.57 had more retrieved and mature oocytes than OS/FP < 0.57, and OS/FP of 0.67 to 0.77 achieved the most (adjusted RR = 1.11, 95% confidence interval [CI]: 1.07–1.15, p for trend = 0.001). In a stratified analysis of age and ovarian stimulation type, OS/FP had a significant effect on the number of retrieved and mature oocytes in women of different ages and ovarian stimulation types (p for trend < 0.001) (**Supplementary Tables 3**, **4**).

**Table 3:** Associations of OS/FP with ovarian response (N = 6110).

	Number of retrieved oocytes^a^ (n)Adjusted RR (95% CI)	Number of retrieved oocytes (n)Margin mean value (95% CI)	Mature oocytes^a^ (n)Adjusted RR (95% CI)	Mature oocytes (n)Margin mean value (95% CI)
Q1 (<0.57)	Ref	10.83 (10.56, 11.10)	Ref	7.88 (7.65, 8.11)
Q2 (0.57, 0.67)	1.05 (1.02, 1.09)**	11.41 (11.16, 11.67)	1.07 (1.03, 1.12)**	8.45 (8.24, 8.67)
Q3 (0.67, 0.77)	1.11 (1.07, 1.15)**	12.01 (11.69, 12.35)	1.14 (1.09, 1.19)**	8.94 (8.67, 9.22)
Q4 (≥0.77)	1.06 (1.02, 1.10)**	11.44 (11.15, 11.74)	1.07 (1.03, 1.12)**	8.46 (8.22, 8.71)
*P*	<0.001**		<0.001**	

Number of retrieved oocytes and mature oocytes were analysed using generalized linear models with poisson distribution and log-linear model.

^a^Models were adjusted for maternal age, BMI, fertility status, etiology of infertility, AMH, type of ovarian stimulation, and gonadotropin initial dose.

*P < 0.05, **P < 0.01. The P value is for the overall trend.

Embryos from 3217 couples continued to be cultured, and a total of 2812 couples had embryos forming blastocysts. The relationship between OS/FP and fertilization rate as well as embryo development is shown in **Table 4**. Fertilization rate were higher in OS/FP of 0.67 to 0.77 than in OS/FP < 0.57 (adjusted OR=1.11, 95% CI: 1.06–1.17, p for trend=0.001). In the age-stratified analysis, the effect of OS/FP on fertilization rate was predominantly present in women of 30 to 35 and >35 years of age (adjusted OR=1.11, 95% CI: 1.01–1.20, p for trend=0.022; adjusted OR=1.27, 95% CI: 1.10–1.48, p for trend=0.001) (**Supplementary Table 5**). Moreover, the role of OS/FP on fertilization rate was mainly found in the long GnRH-agonist protocol (**Supplementary Table 6**). Good-quality embryo rate were lower in OS/FP > 0.77 than in OS/FP < 0.57 (adjusted OR=0.90, 95% CI: 0.82–0.98, p for trend=0.013) which only present in women of 30 to 35 years of age (**Supplementary Table 5**). No significant differences were found in blastocyst formation rate among the four groups. High-quality blastocyst formation rate were lower in OS/FP ≥ 0.77 than in OS/FP < 0.57 (adjusted OR=0.84 95% CI: 0.73–0.96, p for trend=0.01) (**Table 4**). The effects of OS/FP on high-quality blastocyst formation rate were mainly present in women between 30 to 35 years of age (**Supplementary Table 5**). The predictive accuracy and discriminatory capacity of nomograms for fertilization and high-quality blastocyst were low (**Supplementary Figures 1–4**). The corresponding C-indexes were 0.571 and 0.574, respectively.

**Table 4:** Adjusted fertilization, day 3 good-quality embryos, blastocyst formation and high-quality blastocyst (95% CI) by quartile of OS/FP (N = 6110). .

	Fertilization (rate)^a^Adjusted OR (95% CI)	Day 3 good-quality embryos (rate)^b^Adjusted OR (95% CI)	Blastocyst formation (rate)^c^Adjusted OR (95% CI)	High-quality blastocyst formation (rate)^d^Adjusted OR (95% CI)
Q1 (< 0.57)	Ref	Ref	Ref	Ref
Q2 (0.57, 0.67)	1.05 (1.00, 1.10)	1.05 (0.99, 1.10)	1.05 (0.98, 1.13)	0.98 (0.87, 1.10)
Q3 (0.67, 0.77)	1.11 (1.06, 1.17)**	1.04 (0.99, 1.11)	0.93 (0.86, 1.00)	0.93 (0.82, 1.07)
Q4 (≥ 0.77)	1.08 (1.02, 1.14)**	0.99 (0.93, 1.04)	0.98 (0.91, 1.06)	0.84 (0.73, 0.96)**
*P*	<0.001**	0.564	0.147	<0.01**

^a^Models were adjusted for maternal age, BMI, fertility status, etiology of infertility, AMH, type of ovarian stimulation gonadotropin initial dose, and IVF/ICSI techniques.

^b^Models were adjusted for maternal age, BMI, smoke, fertility status, type of ovarian stimulation, gonadotropin initial dose, and IVF/ICSI techniques.

^c^Models were adjusted for maternal age, BMI, type of ovarian stimulation, gonadotropin initial dose, and IVF/ICSI techniques.

^d^Models were adjusted for maternal age, BMI, fertility status, type of ovarian stimulation, gonadotropin initial dose, and IVF/ICSI techniques.

A total of 2812 participants had embryos continuously cultured to the blastocyst stage.

*P < 0.05, **P < 0.01. The P value is for the overall trend.

A total of 2753 women had transferred embryos in this fresh cycle. The relationship between OS/FP and pregnancy outcomes is demonstrated in **Table 5**. No significant differences were found in clinical pregnancy rate, live birth rate, and early miscarriage rate among the four groups. OS/FP was not associated with pregnancy outcomes in the stratified analyses of age and ovarian stimulation type (**Supplementary Tables 7, 8**).

**Table 5:** Clinical pregnancy, live birth and early miscarriage (95% CI) by quartile of OS/FP (N = 2683).

	Clinical pregnancy (rate)^a^Adjusted OR (95% CI)	Live birth (rate)^b^Adjusted OR (95% CI)	Early miscarriage (rate)^c^Adjusted OR (95% CI)
Q1 (<0.57)	Ref	Ref	Ref
Q2 (0.57, 0.67)	1.02 (0.81, 1.28)	0.87 (0.69, 1.08)	1.43 (0.91, 2.25)
Q3 (0.67, 0.77)	1.12 (0.88, 1.43)	1.11 (0.87, 1.42)	1.30 (0.79, 2.16)
Q4 (≥0.77)	1.02 (0.82, 1.26)	0.92 (0.73, 1.16)	1.35 (0.84, 2.17)
*P*	0.692	0.989	0.359

a Models were adjusted for maternal age, BMI, E2-HCG, P-HCG, gonadotropin initial dose, IVF/ICSI techniques, and the number of embryos transferred.

b Models were adjusted for maternal age, BMI, gonadotropin initial dose, IVF/ICSI techniques, and the number of embryos transferred.

c Models were adjusted for maternal age and BMI.

A total of 2648 participants tranferred embryos.

*P < 0.05, **P < 0.01. The P value is for the overall trend.

## Discussion

In this study, OS/FP was observed to have an effect on IVF outcomes in women with regular menstrual cycles undergoing IVF for the first time. In particular, women with OS/FP of 0.67 to 0.77 were able to obtain more oocytes, and had the highest rate of fertilization. With regard to embryos, women with OS/FP higher than 0.77 had the lowest high-quality blastocyst formation rate. OS/FP had no correlation with clinical pregnancy rate, live birth rate or miscarriage rate in the first fresh cycles.

Interestingly, the effects of OS/FP on the rate of good-quality embryos and high-quality blastocyst formation are only present in women of 30 to 35 years of age. A woman’s age has a significant effect on ovarian sensitivity, ovarian responsiveness to exogenous gonadotropin, oocyte quality, embryo-related parameters, and IVF outcomes (34–36). Thus, we speculated that such changes would mask the correspondence between the duration of ovarian stimulation and the follicular phase length in the natural state.

Regular menstrual cycles are often considered to allow spontaneous ovulation; this may sometimes be incorrectly interpreted as an indicator of female fertility. As women age, MCL gradually shortens and variability decreases which is a more sensitive indicator of ovarian aging than physiological age (11). As ovaries age, the resting pool of primordial ovarian follicles gradually diminishes, the cohort of recruited growing follicles declines, and the secretion of inhibin B by granulosa cells decreases, leading to a higher secretion of FSH and an earlier onset of follicular development; thus, ovulation occurs earlier and the follicular phase is shorter.

Predicting ovarian responsiveness to gonadotropin is one of the most important procedures in ART treatment which can optimise the success of treatment. AFC in combination with AMH is generally accepted as a useful method to assess ovarian responsiveness (9, 10). However, in clinical practice, patients with the same AMH levels have exhibited varying ovarian responsiveness. The ovarian response to hormone stimulation varies considerably among women with an AMH level below 1.1 ng/mL (low ovarian responders) and is strongly correlated with MCL (12). Recent evidence demonstrated that long MCLs are related to more antral follicular waves and higher ovarian responses (14, 15). Conversely, short MCLs are associated with poor responses to ovarian stimulation, a marker of ovarian aging (13). Furthermore, MCLs are associated with the number of retrieved oocytes and clinical pregnancy rate in IVF (11), whereas AMH cannot be used as a predictor of oocyte quality or clinical pregnancy outcomes (37). Therefore, MCL is likely to be a better biomarker than AMH for predicting the ovarian response. According to our results, clinicians can estimate the range of ovarian stimulation duration by calculating the original length of the follicular phase to obtain more mature oocytes. In addition, when the duration of ovarian stimulation exceeded 0.77 fold of the original follicular phase length, it may lead to a decrease in embryo quality. The potential underlying mechanism was probably that exposure to high doses of gonadotropins caused chromosomal abnormalities in oocytes and increased the aneuploidy rate of granulosa cells (38, 39).

Previous publications revealed conflicting results when investigating the relationship between the duration of ovarian stimulation and IVF outcomes. Although some studies support the association between prolonged gonadotropin stimulation and poor pregnancy outcome (17, 20, 22), some argue that there is no connection (15, 24), while some have obtained negative correlations (23). The lack of consensus is likely due to a lack of standardized cut-off points for ovulation duration, a small sample size, or failure to correct for confounding factors other than age. The studies which suggest that a longer ovarian stimulation duration is associated with lower live birth rate defined a long ovarian stimulation duration as one that is longer than 13 days, which represents a very small proportion of women, thereby increasing the probability of a positive result.

A major strength of our study is that we demonstrated the possibility of predicting the duration of ovarian stimulation by the MCL. MCL is easy to obtain and does not require any invasive tests, the duration of ovarian stimulation can be individually adjusted according to MCL. Furthermore, In contrast to other studies that used 8 and 12 days as time points for ovarian stimulation, our study limited the optimal choice to a fluctuating range of 1-2 days.

However, this study has several limitations. Only the first fresh cycles were considered in this study. Although we did not observe an effect of OS/FP on clinical pregnancy and live birth rates, previous studies have demonstrated that access to more mature oocytes and high quality embryos improved cumulative pregnancy rates (40, 41). In addition, clinicians may freeze all embryos in fresh cycles because of the moderate or severe ovarian hyper-stimulation syndrome (OHSS), elevated progesterone levels on the day of hCG trigger, low oocyte acquisition, or poor embryo quality (42). Women with extremely high or low OS/FP were subsequently less prone to undergo fresh embryo transfer, and this bias was relatively large, which may lead to the failure to determine the effect of OS/FP on clinical outcomes.

Recent studies have found that the live birth rate in fresh cycles increases with an increasing number of retrieved oocytes, with a plateau occurring when the number of retrieved oocytes reaches 15 or more, and the cumulative live birth rates for both fresh and frozen cycles consistently increase with an increasing number of retrieved oocytes, without a plateau (40, 43–45). The incidence of severe OHSS also increases with an increasing number of retrieved oocytes, especially when the number of retrieved oocytes reaches 18 and above, and the incidence of thromboembolism also increases significantly when the number of retrieved oocytes is more than 15 (46). In this study, women with OS/FP of 0.67 to 0.77 had access to more oocytes, but when the number of follicles (> 14 mm) reached 15 and above, an appropriate reduction in the duration of ovarian stimulation was considered depending on the length of the follicular phase to reduce the possibility of OHSS and thromboembolism. In addition, when patients have a diminished capacity for oocyte and embryo development, clinicians are required to optimize the ovarian response to gonadotropin by accurately estimating the ovarian reserve to customize an appropriate strategy to collect the maximum number of oocytes (47). Therefore, when the number of follicles (> 14 mm) is below or equal to 5, it may be beneficial to optimize the duration of ovarian stimulation to obtain more oocytes depending on the length of the follicular phase.

Overall, we present a new clinically valuable parameter, OS/FP, which can affect the number of retrieved oocytes, fertilization rate, and embryo quality. The trigger time can be easily optimized by referring to the length of the woman’s original follicular phase. Future studies will need to explore the role of OS/FP in ART through further expansion of the sample size and interventional studies to reveal the underlying mechanisms and consequently provide guidance for clinicians to optimize the appropriate trigger timing.

## Data Availability Statement

The raw data supporting the conclusions of this article will be made available by the authors, without undue reservation.

## Ethics Statement

The studies involving human participants were reviewed and approved by the Ethics Committee of the Shengjing Hospital of China Medical University (2021PS001F). Written informed consent for participation was not required for this study in accordance with the national legislation and the institutional requirements.

## Author Contributions

XiZ: Design, conception, analysis and interpretation of data, drafting of article, visualization. XuZ: Acquisition of data, design, drafting of article. SSW: Analysis and interpretation of data, writing - review & editing, visualization. JCT: Conception, resources, writing - review & editing, supervision, funding acquisition. All authors approved the final manuscript. All authors contributed to the article and approved the submitted version.

## Funding

The work was supported by the National Key Research and Development Program (2018YFC1002105), the National Natural Science Foundation of China (82071601, 61873257), the Shengjing Freelance Researcher Plan of Shengjing Hospital of China Medical University and the Major Special Construction Plan for Discipline Construction Project of China Medical University (3110118033).

## Conflict of Interest

The authors declare that the research was conducted in the absence of any commercial or financial relationships that could be construed as a potential conflict of interest.

## Publisher’s Note

All claims expressed in this article are solely those of the authors and do not necessarily represent those of their affiliated organizations, or those of the publisher, the editors and the reviewers. Any product that may be evaluated in this article, or claim that may be made by its manufacturer, is not guaranteed or endorsed by the publisher.
