# Supplementary material for: Association Between the Ratio of Ovarian Stimulation Duration to Original Follicular Phase Length and In Vitro Fertilization Outcomes: A Novel Index to Optimise Clinical Trigger Time
Source: Front Endocrinol (Lausanne). 2022 Jul 25;13:862500. doi: 10.3389/fendo.2022.862500 (PMC9361069; doi:10.3389/fendo.2022.862500)
Supplement: Supplementary file 1 [file DataSheet_1.docx]

Supplementary Material

# Supplementary Figures and Tables

## Supplementary Figures


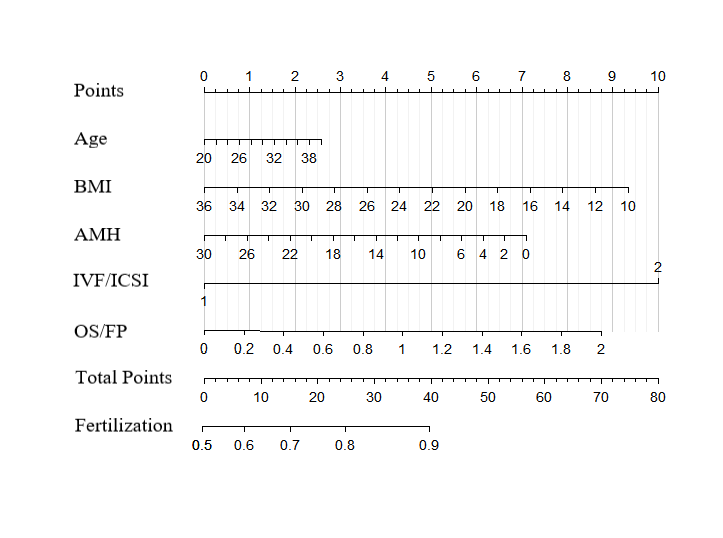


**Supplementary Figure 1.** Nomogram for fertilization.


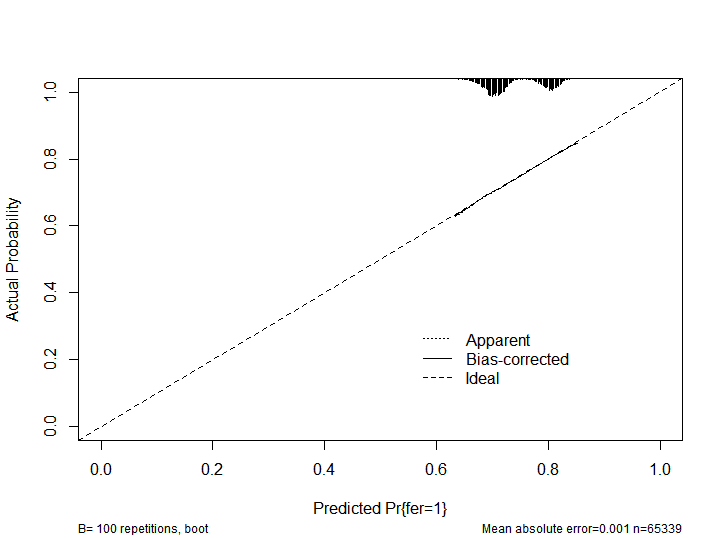


**Supplementary Figure 2**. Calibration curve of nomogram for fertilization.


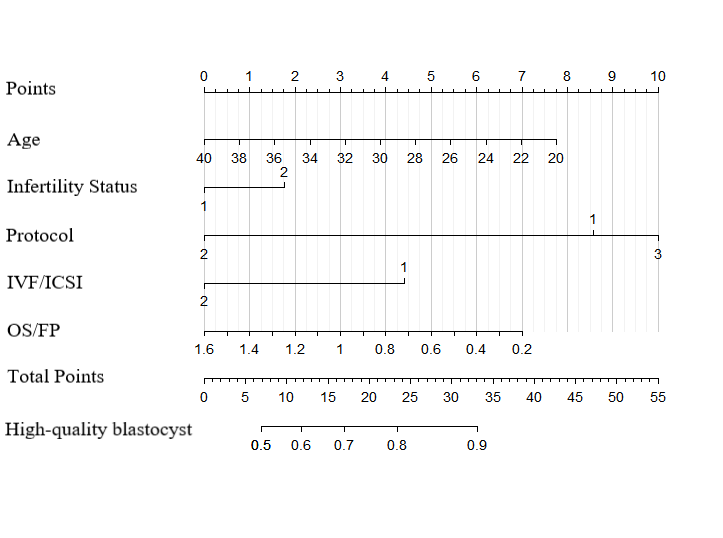


**Supplementary Figure 3.** Nomogram for high-quality blastocyst.


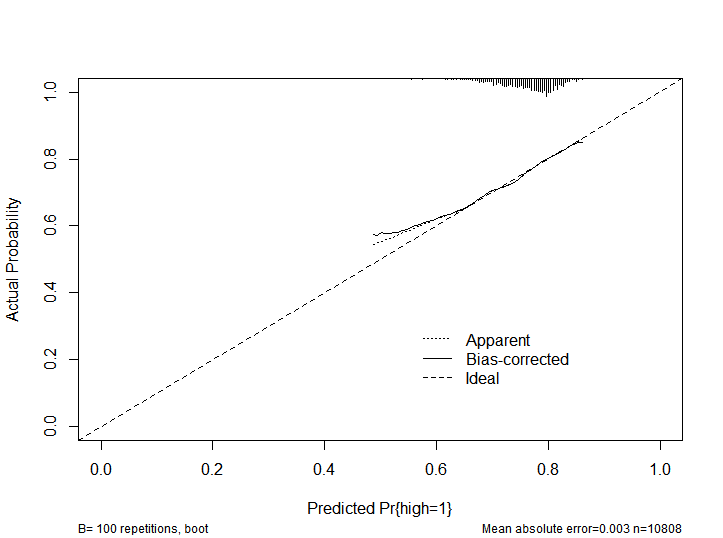


**Supplementary Figure 4.** Calibration curve of nomogram for high-quality blastocyst.

## Supplementary Tables

**Supplementary Table 1 The distributions of OS/FP in this study.**

|  | **Mutiple Value** |
| --- | --- |
| Mean | 0.68 |
| SD | 0.16 |
| Min | 0.13 |
| Median | 0.67 |
| Max | 1.88 |
| IQR | (0.56, 0.75) |

Abbreviations: SD: standard deviation; Min: minimum; Max: maximum; IQR, interquartile range.

**Supplementary Table 2 Demographic and clinical characteristics of all the participants (N = 6110).**

| **Item** | **Value** |
| --- | --- |
| N | 6110 |
| Age (years) | 31.5±3.9 |
| ≤30, n(%) | 2520(41.2) |
| 30-35, n(%) | 2567(42.0) |
| >35, n(%) | 1023(16.7) |
| BMI (kg/m2 ) | 22.7±3.3 |
| BMI (kg/m2 ) category^a^ |  |
| Underweight (<18.5) | 461(7.5) |
| Normal (18.5–24.9) | 4138(67.7) |
| Overweight (25.0–29.9) | 1222(20) |
| Obese (≥30.0) | 180(2.9) |
| AMH (ng/ml) | 3.8±2.6 |
| Gravidity |  |
| 0 | 3363(55.0) |
| 1+ | 2747(45.0) |
| Parity |  |
| Nulliparous | 5674(92.9) |
| 1+ | 436(7.1) |
| Abortions |  |
| 0 | 4091(67.0) |
| 1+ | 2019(33.0) |
| Menstrual cycle length (days) | 29.0±2.0 |
| Usual menstrual bleeding (days) | 5.2±1.4 |
| Age at first menstruation (years) | 13.6±1.2 |
| Duration of infertility (years) | 3.7±2.7 |
| Bachelor or above | 2391(39.1) |
| Current Smoker | 102(1.6) |
| Fertility status |  |
| Primary infertility | 3686(60.3) |
| Secondary infertility | 2424(39.7) |
| Etiology of infertility |  |
| Female factor | 3119(51.0) |
| Male factor | 1276(20.9) |
| Mixed factors | 1506(24.6) |
| Idiopathic | 209(3.4) |
| Type of ovarian stimulation |  |
| Long GnRH-agonist | 3196(52.3) |
| Short GnRH-agonist | 243(4.0) |
| GnRH-antagonist | 2671(43.7) |
| Initial gonadotropin dose, IU | 228.2±54.2 |
| Total gonadotropin dose, IU | 2359.2±827.5 |
| Endometrial thickness-HCG, mm | 10.2±2.8 |
| Duration of stimulation (days) | 9.9±2.0 |
| Insemination technique |  |
| IVF | 3831(62.7) |
| ICSI | 2279(37.3) |

Values are n (%) for categorical variables and mean ± SD for continuous variables.

Abbreviations: SD, standard deviation; BMI, body mass index; AMH, anti-mullerian hormone; IVF, in vitro fertilization; ICSI, intracytoplasmic sperm injection.

Data on covariates were missing for BMI (n = 109), endometrial thickness-HCG (n=99).

^a^BMI category (WHO classifification).

**Supplementary Table 3 Associations of OS/FP with ovarian response stratified by age (N = 6110).**

|  | **Number of retrieved oocytes^a^ (n)**  **Adjusted RR (95% CI)** | **Number of retrieved oocytes (n)**  **Margin mean value (95% CI)** | **Mature oocytes^a^ (n)**  **Adjusted RR (95% CI)** | **Mature oocytes (n)**  **Margin mean value (95% CI)** |
| --- | --- | --- | --- | --- |
| **Age≤30 (n=2520)** | | | | |
| **Q1(<0.57)** | Ref | 12.34 (11.89, 12.81) | Ref | 9.05 (8.68, 9.44) |
| **Q2(0.57, 0.67)** | 1.03 (0.98, 1.09)* | 12.74 (12.32, 13.17) | 1.05 (0.99, 1.11) | 9.51 (9.15, 9.89) |
| **Q3(0.67, 0.77)** | 1.10 (1.04, 1.17)** | 13.56 (13.00, 14.15) | 1.11 (1.04, 1.18)** | 10.06 (9.61, 10.54) |
| **Q4 (≥0.77)** | 1.06 (1.00, 1.12)** | 13.04 (12.54, 13.55) | 1.05 (0.99, 1.12) | 9.52 (9.1, 9.95) |
| **P** | <0.05* |  | <0.05* |  |
| **Age(30,35) (n=2567)** | | | | |
| **Q1(<0.57)** | Ref | 10.46 (10.08, 10.85) | Ref | 7.57 (7.24, 7.91) |
| **Q2(0.57, 0.67)** | 1.06 (1.01, 1.11)* | 11.07 (10.71, 11.45) | 1.07 (1.01, 1.14)* | 8.12 (7.81, 8.45) |
| **Q3(0.67, 0.77)** | 1.11 (1.05, 1.17)** | 11.57 (11.10, 12.06) | 1.14 (1.07, 1.22)** | 8.66 (8.27, 9.07) |
| **Q4 (≥0.77)** | 1.05 (0.99, 1.12) | 11.01 (10.57, 11.48) | 1.09 (1.02, 1.16)** | 8.25 (7.89, 8.63) |
| **P** | <0.01** |  | <0.001** |  |
| **Age>35 (n=1023)** | | | | |
| **Q1(<0.57)** | Ref | 8.33 (7.80, 8.90) | Ref | 5.93 (5.49, 6.4) |
| **Q2(0.57, 0.67)** | 1.08 (1.02, 1.14) | 8.99 (8.49, 9.51) | 1.13 (1.02, 1.25)* | 6.68 (6.26, 7.13) |
| **Q3(0.67, 0.77)** | 1.18 (1.08, 1.29)** | 9.82 (9.22, 10.47) | 1.23 (1.10, 1.37)** | 7.27 (6.71, 7.87) |
| **Q4 (≥0.77)** | 1.08 (1.01, 1.14) | 8.98 (8.45, 9.55) | 1.14 (1.02, 1.27)* | 6.72 (6.25, 7.23) |
| **P** | <0.01** |  | <0.01** |  |

|  |
| --- |

Number of retrieved oocytes and mature oocytes were analysed using generalized linear models with poisson distribution and log-linear model.

^a^Models were adjusted for maternal age, BMI, fertility status, etiology of infertility, AMH, type of ovarian stimulation， and gonadotropin initial dose.

**P* < 0.05, ***P*< 0.01. The *P* value is for the overall trend.

**Supplementary Table 4 Associations of OS/FP with ovarian response stratified by ovarian stimulation type (N = 5867).**

|  | **Number of retrieved oocytes^a^ (n)**  **Adjusted RR (95% CI)** | | **Number of retrieved oocytes (n)**  **Margin mean value (95% CI)** | **Mature oocytes^a^ (n)**  **Adjusted RR (95% CI)** | | **Mature oocytes (n)**  **Margin mean value (95% CI)** | |
| --- | --- | --- | --- | --- | --- | --- | --- |
| **Long GnRH-agonist (n=3196)** | | | | | | | |
| **Q1(<0.57)** | | Ref | 11.32 (10.88, 11.77) | | Ref | | 8.08 (7.71, 8.47) |
| **Q2(0.57, 0.67)** | | 1.08 (1.02, 1.13)** | 12.17 (11.8, 12.55) | | 1.10 (1.04, 1.17)** | | 8.88 (8.56, 9.21) |
| **Q3(0.67, 0.77)** | | 1.13 (1.07, 1.19)** | 12.82 (12.39, 13.26) | | 1.18 (1.11, 1.26)** | | 9.54 (9.19, 9.90) |
| **Q4 (≥0.77)** | | 1.08 (1.03, 1.14)** | 12.21 (11.84, 12.59) | | 1.11 (1.05, 1.18)** | | 8.96 (8.65, 9.28) |
| **P** | | <0.001** |  | | <0.001** | |  |
| **GnRH-antagonist (n=2671)** | | | | | | | |
| **Q1(<0.57)** | | Ref | 10.32 (10.00, 10.65) | | Ref | | 7.69 (7.42, 7.97) |
| **Q2(0.57, 0.67)** | | 1.04 (0.99, 1.09) | 10.72 (10.38, 11.08) | | 1.05 (1.00, 1.11)* | | 8.10 (7.80, 8.41) |
| **Q3(0.67, 0.77)** | | 1.09 (1.03, 1.16)** | 11.30 (10.75, 11.87) | | 1.08 (1.01, 1.16)* | | 8.33 (7.89, 8.80) |
| **Q4 (≥0.77)** | | 1.03 (0.97, 1.09) | 10.64 (10.14, 11.17) | | 1.04 (0.97, 1.11) | | 7.97 (7.55, 8.40) |
| **P** | | <0.05* |  | | 0.073 | |  |

Number of retrieved oocytes and mature oocytes were analysed using generalized linear models with poisson distribution and log-linear model.

^a^Models were adjusted for maternal age, BMI, fertility status, etiology of infertility, AMH, and gonadotropin initial dose.

**P* < 0.05, ***P*< 0.01. The *P* value is for the overall trend.

**Supplementary Table 5 Associations of OS/FP with embryonic developmental parameters stratified by age (N=6110).**

|  | **Fertilization (rate)^a^**  **Adjusted OR (95% CI)** | **Day 3 good-quality embryos (rate)^b^**  **Adjusted OR (95% CI)** | **Blastocyst formation (rate)^c^**  **Adjusted OR (95% CI)** | **High-quality blastocyst formation (rate)^d^**  **Adjusted OR (95% CI)** |
| --- | --- | --- | --- | --- |
| **Age≤30 (n=2520)** | | | | |
| **Q1(<0.57)** | Ref | Ref | Ref | Ref |
| **Q2(0.57, 0.67)** | 1.06 (0.99, 1.14) | 1.11 (1.03, 1.20)** | 1.05 (0.95, 1.16) | 1.00 (0.84, 1.20) |
| **Q3(0.67, 0.77)** | 1.08 (1.01, 1.17)* | 1.08 (1.00, 1.18) | 0.86 (0.76, 0.96)** | 0.77 (0.64, 0.94)* |
| **Q4 (≥0.77)** | 1.05 (0.98, 1.14) | 1.06 (0.97, 1.15) | 0.95 (0.85, 1.07) | 0.89 (0.73, 1.09) |
| ***P*** | 0.128 | 0.294 | 0.047* | 0.045* |
| **Age(30,35) (n=2567)** | | | | |
| **Q1(<0.57)** | Ref | Ref | Ref | Ref |
| **Q2(0.57, 0.67)** | 1.01 (0.94, 1.08) | 1.02 (0.94, 1.10) | 1.07 (0.96, 1.19) | 0.93 (0.78, 1.12) |
| **Q3(0.67, 0.77)** | 1.11 (1.02, 1.20)* | 1.00 (0.92, 1.10) | 0.93 (0.83, 1.05) | 1.09 (0.88, 1.33) |
| **Q4 (≥0.77)** | 1.07 (0.99, 1.16) | 0.90 (0.82, 0.98)** | 0.91 (0.81, 1.04) | 0.73 (0.60, 0.89)** |
| ***P*** | 0.022* | 0.013* | 0.051 | 0.022* |
| **Age>35 (n=1023)** | | | | |
| **Q1(<0.57)** | Ref | Ref | Ref | Ref |
| **Q2(0.57, 0.67)** | 1.17 (1.02, 1.34)* | 0.99 (0.85, 1.15) | 0.89 (0.72, 1.11) | 0.99 (0.70, 1.41) |
| **Q3(0.67, 0.77)** | 1.27 (1.10, 1.48)** | 1.07 (0.91, 1.26) | 1.02 (0.81, 1.29) | 0.97 (0.67, 1.42) |
| **Q4 (≥0.77)** | 1.25 (1.09, 1.44)** | 1.04 (0.89, 1.21) | 1.05 (0.83, 1.31) | 0.82 (0.57, 1.18) |
| ***P*** | <0.01** | 0.431 | 0.410 | 0.258 |

^a^Models were adjusted for maternal age, BMI, fertility status, etiology of infertility, AMH, type of ovarian stimulation, gonadotropin initial dose, and IVF/ICSI techniques.

^b^Models were adjusted for maternal age, BMI, smoke, fertility status, AMH, type of ovarian stimulation, gonadotropin initial dose, and IVF/ICSI techniques.

^c^Models were adjusted for maternal age, BMI, type of ovarian stimulation, gonadotropin initial dose, and IVF/ICSI techniques.

^d^Models were adjusted for maternal age, BMI, fertility status, type of ovarian stimulation, gonadotropin initial dose, and IVF/ICSI techniques.

A total of 2812 participants had embryos continuously cultured to the blastocyst stage.

**P* < 0.05, ***P*< 0.01. The *P* value is for the overall trend.

**Supplementary Table 6 Associations of OS/FP with embryonic developmental parameters stratified by ovarian stimulation type (N = 5867).**

|  | **Fertilization (rate)^a^**  **Adjusted OR (95% CI)** | **Day 3 good-quality embryos (rate)^b^**  **Adjusted OR (95% CI)** | **Blastocyst formation (rate)^c^**  **Adjusted OR (95% CI)** | **High-quality blastocyst formation^d^**  **Adjusted OR (95% CI)** |
| --- | --- | --- | --- | --- |
| **Long GnRH-agonist (n=3196)** | | | | |
| **Q1(<0.57)** | Ref | Ref | Ref | Ref |
| **Q2(0.57, 0.67)** | 0.99 (0.93, 1.07) | 0.98 (0.90, 1.06) | 1.14 (1.02, 1.27)* | 1.17 (0.98, 1.41) |
| **Q3(0.67, 0.77)** | 1.14 (1.06, 1.22)** | 1.01 (0.93, 1.10) | 1.00 (0.89, 1.12) | 1.03 (0.85, 1.24) |
| **Q4 (≥0.77)** | 1.06 (0.98, 1.13) | 0.95 (0.88, 1.03) | 1.06 (0.94, 1.19) | 0.96 (0.80, 1.16) |
| ***P*** | <0.01** | 0.349 | 0.948 | 0.229 |
| **GnRH-antagonist (n=2671)** | | | | |
| **Q1(<0.57)** | Ref | Ref | Ref | Ref |
| **Q2(0.57, 0.67)** | 1.09 (1.02, 1.16)* | 1.14 (1.06, 1.23)** | 1.02 (0.93, 1.12) | 0.92 (0.78, 1.08) |
| **Q3(0.67, 0.77)** | 1.04 (0.96, 1.12) | 1.07 (0.98, 1.17) | 0.86 (0.77, 0.97)* | 0.90 (0.74, 1.10) |
| **Q4 (≥0.77)** | 1.09 (1.01, 1.19)* | 1.04 (0.95, 1.14) | 0.92 (0.81, 1.04) | 0.73 (0.60, 0.89)** |
| ***P*** | 0.066 | 0.346 | 0.029* | <0.01** |

^a^Models were adjusted for maternal age, BMI, fertility status, etiology of infertility, AMH, gonadotropin initial dose, and IVF/ICSI techniques.

^b^Models were adjusted for maternal age, BMI, smoke, fertility status, AMH, gonadotropin initial dose, and IVF/ICSI techniques.

^c^Models were adjusted for maternal age, BMI, gonadotropin initial dose, and IVF/ICSI techniques.

^d^Models were adjusted for maternal age, BMI, fertility status, gonadotropin initial dose, and IVF/ICSI techniques.

A total of 2812 participants had embryos continuously cultured to the blastocyst stage.

**P* < 0.05, ***P*< 0.01. The *P* value is for the overall trend.

**Supplementary Table 7 Associations of OS/FP with pregnancy outcomes stratified by age (N=2648).**

|  | **Clinical pregnancy (rate)^a^**  **Adjusted OR (95% CI)** | **Live birth (rate)^b^**  **Adjusted OR (95% CI)** | **Early miscarriage (rate)^c^**  **Adjusted OR (95% CI)** |
| --- | --- | --- | --- |
| **Age≦ 30 (N=1044)** | | | |
| **Q1(<0.57)** | Ref | Ref | Ref |
| **Q2(0.57, 0.67)** | 1.06 (0.75, 1.50) | 0.82 (0.58, 1.16) | 1.31 (0.66, 2.63) |
| **Q3(0.67, 0.77)** | 1.11 (0.76, 1.63) | 1.03 (0.71, 1.50) | 1.12 (0.54, 2.34) |
| **Q4 (≥0.77)** | 1.31 (0.91, 1.88) | 0.99 (0.69, 1.42) | 0.48 (0.19, 1.19) |
| ***P*** | 0.146 | 0.712 | 0.131 |
| **Age (30, 35) (N=1149)** | | | |
| **Q1(<0.57)** | Ref | Ref | Ref |
| **Q2(0.57, 0.67)** | 0.79 (0.57, 1.09) | 0.77 (0.56, 1.07) | 0.85 (0.46, 1.56) |
| **Q3(0.67, 0.77)** | 1.00 (0.75, 1.58) | 1.12 (0.78, 1.62) | 0.95 (0.48, 1.89) |
| **Q4 (≥0.77)** | 0.72 (0.50, 1.02) | 0.76 (0.53, 1.08) | 0.83 (0.43, 1.62) |
| ***P*** | 0.235 | 0.427 | 0.690 |
| **Age > 35 (N=455)** | | | |
| **Q1(<0.57)** | Ref | Ref | Ref |
| **Q2(0.57, 0.67)** | 1.75 (0.98, 3.12) | 1.41 (0.76, 2.64) | 2.56 (0.78, 8.35) |
| **Q3(0.67, 0.77)** | 1.26 (0.67, 2.35) | 1.36 (0.70, 2.64) | 1.18 (0.28, 4.88) |
| **Q4 (≥0.77)** | 1.55 (0.88, 2.73) | 1.37 (0.74, 2.52) | 2.20 (0.68, 7.16) |
| ***P*** | 0.332 | 0.424 | 0.437 |

^a^Models were adjusted for maternal age, BMI, gonadotropin initial dose, E2-HCG, P-HCG, IVF/ICSI techniques, the number of embryos transferred .

^b^Models were adjusted for maternal age, BMI, gonadotropin initial dose, IVF/ICSI techniques and the number of embryos transferred.

^c^Models were adjusted for maternal age, BMI.

A total of 2648 participants tranferred embryos.

**P* < 0.05, ***P*< 0.01. The *P* value is for the overall trend.

**Supplementary Table 8 Associations of OS/FP with pregnancy outcomes stratified by ovarian stimulation type (N=2648).**

|  | **Clinical pregnancy (rate)^a^**  **Adjusted OR (95% CI)** | **Live birth (rate)^b^**  **Adjusted OR (95% CI)** | **Early miscarriage (rate)^c^**  **Adjusted OR (95% CI)** |
| --- | --- | --- | --- |
| **Long GnRH-agonist (N=1455)** | | | |
| **Q1(<0.57)** | Ref | Ref | Ref |
| **Q2(0.57, 0.67)** | 1.24 (0.90, 1.71) | 0.95 (0.68, 1.31) | 1.68 (0.84, 3.34) |
| **Q3(0.67, 0.77)** | 1.22 (0.87, 1.71) | 1.12 (0.80, 1.59) | 1.66 (0.81, 3.44) |
| **Q4 (≥0.77)** | 1.30 (0.94, 1.78) | 1.16 (0.84, 1.59) | 1.38 (0.68, 2.78) |
| ***P*** | 0.178 | 0.184 | 0.626 |
| **GnRH-antagonist (N=1034)** | | | |
| **Q1(<0.57)** | Ref | Ref | Ref |
| **Q2(0.57, 0.67)** | 0.93 (0.68, 1.28) | 0.79 (0.57, 1.09) | 1.30 (0.68, 2.49) |
| **Q3(0.67, 0.77)** | 1.20 (0.82, 1.76) | 1.13 (0.78, 1.65) | 1.15 (0.51, 2.57) |
| **Q4 (≥0.77)** | 0.79 (0.53, 1.18) | 0.56 (0.37, 0.86)** | 1.32 (0.60, 2.90) |
| ***P*** | 0.601 | 0.073 | 0.548 |

^a^Models were adjusted for maternal age, BMI, gonadotropin initial dose, E2-HCG, P-HCG, IVF/ICSI techniques, the number of embryos transferred .

^b^Models were adjusted for maternal age, BMI, gonadotropin initial dose, IVF/ICSI techniques and the number of embryos transferred.

^c^Models were adjusted for maternal age, BMI.

A total of 2648 participants tranferred embryos.

**P* < 0.05, ***P*< 0.01. The *P* value is for the overall trend.
